# Bone marrow mesenchymal stroma cell serves as a harbor anchoring acute B lymphoblastic leukemia cells

**DOI:** 10.1016/j.gendis.2025.101637

**Published:** 2025-04-12

**Authors:** Kunpeng Wu, Fen Wang, Min Shen, Xiaohui Liang, Dan Wei, Xi Zhu, Ying Qu, Xunbin Wei, Hua Jiang, Tong Chen

**Affiliations:** a Department of Hematology, Huashan Hospital, Fudan University, Shanghai 200040, China; b Center of Precision Medicine for Blood Diseases, Fudan University, Shanghai 200040, China; c Department of Rehabilitation, Shanghai General Hospital, Shanghai Jiaotong University, Shanghai 200240, China; d Med-X Research Institute and School of Biomedical Engineering, Shanghai Jiao Tong University, Shanghai 200030, China; e Peking University Cancer Hospital & Institute, Beijing 100142, China; f Biomedical Engineering Department, Peking University, Beijing 100081, China; g Institute of Medical Technology, Peking University Health Science Center, Beijing 100191, China; h International Cancer Institute, Peking University, Beijing 100191, China; i Obstetrics & Gynecology Hospital, Fudan University, Shanghai 200011, China

A growing body of evidences suggests that mesenchymal stroma cells (MSCs) are not only a therapeutic resource to treat various diseases, but also an important pathogenic worker in tumorigenesis.^1^ However, the differences between *in vitro* and *in vivo* environment draw the major concern leading to the contradictory effects of MSCs on tumorigenesis.^2^ Technical limitation makes it even hard to dissect the real cell–cell interacting model in a living status. As like a two-edge sword, dissecting the underlying mechanism resulting in this dilemma would help to apply MSCs more accurately in translational medicine. In our study, we applied murine *in vivo* calvarium intravital microscopy (IVM) and *in vivo* flow cytometry (IVFC) to directly observe the dynamic cell–cell interaction between acute lymphoblastic leukemia cells (L1210s) and BM (bone marrow)-MSCs. Our data clarified a living model by which BM-MSCs provide a micro-niche to facilitate leukemia cells invasion.

To clarify the living interaction between leukemia cells and externally administered MSCs, the distribution of DiD-labelling L1210 cells and Dil-labelling BM-MSCs were detected on our IVFC and IVM platform. Interestingly, even though L1210 cells and BM-MSCs were injected separately at a 20-min interval, a co-localized lodging manner of both types of cells was found either in live peripheral circulation or in intravital BM. By using IVFC, the overlay of DID-L1210 signaling burst and Dil-MSC burst was detected as the co-existence where only DID signaling was showed in L1210-injected group (Fig. S1A). The overlaid bursts reached 10.9% of L1210 cells 1 h post-transplantation, where only 1.2% overlaid bursts were investigated in the co-injection of DiD-L1210 cells with Dil-L1210 cells (Fig. S1B). By IVM investigation, L1210s and MSCs were distributed closely overall in skull marrow after transplantation (Fig. 1A). About 28% of injected L1210 cells within BM were co-localized with MSCs, whose percentage was much higher than that of L1210 cells with random dots (Fig. 1B). The results of overlaying L1210-MSC bursts in PB and relatively increased co-localizing percentage in BM indicate that the co-localization of L1210 cells and BM-MSCs are not resulted from random cellular distribution.

**Figure 1:**
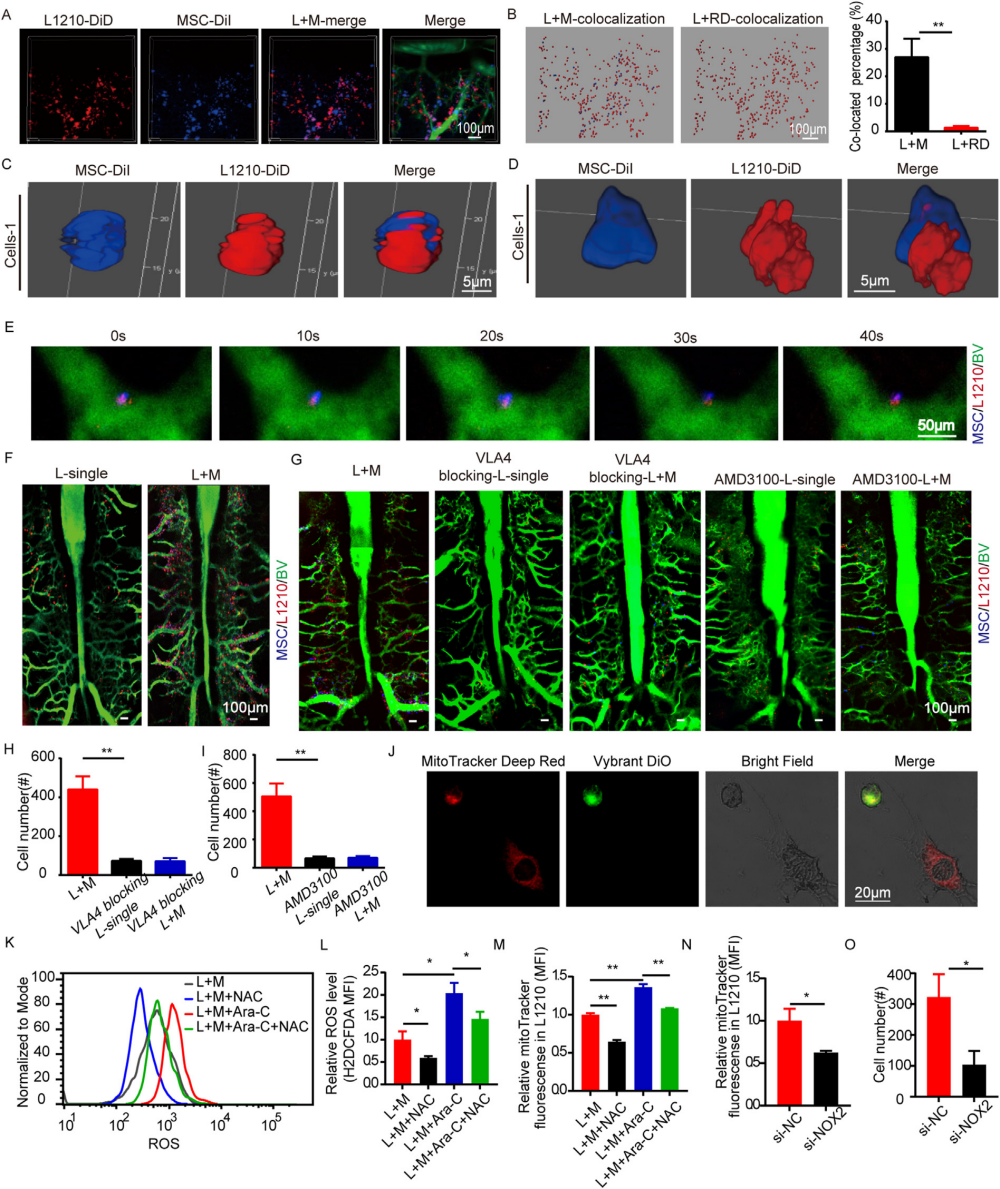
MSC promoted the homing and and invasion of L1210 required the tight adhesion of L1210 with MSCs, probably through L1210 drived mitochondrial transfer. **(A)** L1210s and MSCs distributed closely in skull marrow after transplantation. Red: L1210s labeled by DiD. Blue: MSC labeled by DiI. Green: bone marrow vessels labeled by FITC-dextran. **(B)** The difference in co-located dots number between L1210 + MSC and L1210 + RD in skull marrow (*n* = 5). White: inserted RDs. **(C)** The imaging of L1210 + MSC co-localizing cells in PB under confocal. Red: L1210s labeled by DiD. Blue: MSC labeled by DiI. **(D)** The imaging of L1210 + MSC co-localizing cells in BM of both hind limbs under confocal. Red: L1210s labeled by DiD. Blue: MSC labeled by DiI. **(E)** L1210 and MSC co-localizing cells acrossed the vessel together in the skull BM. Red: L1210 labeled by DiD. Blue: MSC labeled by DiI. Green: bone marrow vessels labeled by FITC dextran. **(F)** The *in vivo* imaging of the homing L1210s in skull marrow in L1210 transplantation group and L1210 + MSC co-transplantation group. Red: L1210s labeled by DiD. Blue: MSC labeled by DiI. Green: bone marrow vessels labeled by FITC-dextran. **(G)** The *in vivo* imaging of the homing L1210s in skull marrow in L1210-MSC, VLA-4 blocking L1210, VLA-4 blocking L1210-MSC, CXCR4 blocking L1210 and CXCR4 blocking L1210-MSC transplantation group. Red: L1210s labeled by DiD. Blue: MSC labeled by DiI. Green: bone marrow vessels labeled by FITC-dextran. **(H–I)** The statistic analysis of the homing L1210 cell number in the bone marrow per field (*n* = 4). **(J)** Live cell confocal imaging of MSCs stained with Deep MitoTracker Red (Red) in co-culture with L1210 cells (green) stained with DiO. **(K–L)** Modulated the ROS levels with NAC (5 mM), Ara-c (200 nM), or NAC (5 mM) and Ara-c (200 nM) together. Typical graphs (K) and statistical comparisons (L) of ROS levels were displayed in MSCs. **(M)** The relative Mitotracker fluorescence in L1210s with NAC (5 mM), Ara-c (200 nM), or NAC (5 mM) and Ara-c (200 nM) treatment after coculture with MSCs determined by flow cytometry analysis (*n* = 3). **(N)** MitoTracker MFI levels in si-NOX2 or si-NC L1210s after coculture with MSCs for 24 h (*n* = 3). **(O)** The statistic analysis of the homing si-NOX2 or si-NC L1210 cell number in the bone marrow per field (*n* = 3).

To further verify the status of cell–cell contact between L1210 cells and BM-MSCs, we investigated the adherent cells under confocal laser scanning microscopy (CLSM) to detect their contacting profile. Surprisingly, a much tight junction as cross hinge showing an interwoven style between two cells were detected either in PB (Fig. 1C; Fig. S1C) or in BM (Fig. 1D; Fig. S1D), again indicating that an integrated adherence between leukemia cells and BM-MSCs is an important model for the inter-cell message transfer.

We thus monitored how leukemic cells and injected BM-MSCs link each other in live murine calvarium by IVM. Ten minutes after the cells injected, the co-localization of DiD-L1210 cells and Dil-MSCs was viewed either inside or outside of blood vessels, showing that the transmembrane mobility was happened immediately after the cells being injected (Fig. S2A). A single L1210 cell and a single MSC were found to move towards each other within the vessel at the direction of either MSC to L1210 cell (Fig. S2B, Sup. Video 1) or L1210 cell to MSC (Fig. S2C, Sup. Video 2), and travel together in the vessel (Fig. S2D). Thereafter a timely L1210-MSC contact and attachment was viewed (Fig. S2E, Sup. Video 3). Interestingly, the L1210-MSC integrated cells were showed to across the vessel together (Fig. 1E).

Supplementary video related to this article can be found at https://doi.org/10.1016/j.gendis.2025.101637

Supplementary video related to this article can be found at https://doi.org/10.1016/j.gendis.2025.101637

The following are the supplementary data related to this article.

Multimedia component 2 2 Multimedia component 2

Multimedia component 3 3 Multimedia component 3

Multimedia component 4 4 Multimedia component 4

The *in vitro* dynamic cell–cell micro-interaction was also investigated under CLSM. Mcherry^+^ L1210 cells showed a tendency moving towards and distributed around GFP^+^ MSCs during the culture process (Fig. S3A). Within 24 h, a stable attachment of L1210-MSC, even a cluster of several L1210 cells attached to a single MSC was seen under microscope (Fig. S3B). This type of self-directed movement indicated that there might be an underlying motivator driving leukemia cells and MSCs perform facing motility.

We thus investigated the biological role of externally administered BM-MSCs in L1210 cell homing both *in vitro* and *in vivo*. L1210 cells were co-cultured with BM-MSCs for 24 h, pre-cultivation with BM-MSCs significantly enhanced the transmigrating ability of L1210 cells (Fig. S4A, B). As for *in vivo* experiments, 24 h after cell injection, mcherry^+^ L1210 cells were at 1.21% of total BM cells in co-transplanted mice whereas in L1210-injected group, mcherry^+^ cells were almost half-reduced (Fig. S4C, D). *In vivo* IVM also revealed that intra-BM homed L1210 cells in the sequential co-injection group were much more than those in single L1210 injected groups (Fig. 1F; Fig. S4E, F).

The CXCR4 chemokine receptor and VLA-4 integrins expressed on the B cells are the vital molecules to facilitate the attraction and adhesion of MSCs and B cells.^3^ Then we clarified whether the prompting homing effect of MSC was depended on the chemotaxis and adhesion between L1210 cells and BM-MSCs. The homing L1210 was decreased after VLA-4 (Fig. 1G, H; Fig. S5A) and CXCR4 (Fig. 1G, I; Fig. S5B) blockage on L1210 with MSC-coinjection. These data demonstrated that inhibition of VLA-4 and CXCL4 abrogated the prompting homing effect of MSCs on L1210. The mRNA levels of CXCR4 and VLA-4 showed no difference between L1210 cells cultured alone and cocultured with MSCs (Fig. S5C).

MSCs have been shown to donate their mitochondria to T-ALL.^4^ Notably, MSCs mitochondria transfer relies on tight cell adhesion.^5^ To further identify the complex interactions between L1210s and MSCs, we detected the mitochondrial transfer. After 24 h coculture, MSCs transferred the mitochondria to the L1210 cells as indicated by the red fluorescence of Mitotracker in L1210 cells (Fig. 1J). ROS levels were closely related to cellular activities include mitochondrial transfer. We found that ROS levels in MSCs and L1210 cocultures were increased than monoculture of MSCs, indicating L1210 induced an increase in ROS levels in MSCs (Fig. S6A). To identify the interactions between ROS and mitochondrial transfer, we modulated the ROS levels with NAC (5 mM), Ara-c (200 nM), or NAC (5 mM) and Ara-c (200 nM) together, and the ROS level in MSC were successfully manipulated (Fig. 1K, L). The relative Mitotracker fluorescence in L1210s was remarkably decreased with NAC treatment while remarkably increased with Ara-c treatment (Fig. 1M).

NOX2-derived ROS in AML is responsible for mitochondrial transfer from MSCs.^5^ Therefore, we knocked down NOX2 in L1210 using a siRNA that targeted NOX2 gene (Fig. S6B). We subsequently tested whether L1210 with NOX2 knockdown could influence ROS and in MSCs. Interestingly, the ROS level in MSCs (Fig. S6C) declined after the knocking down of NOX2 in L1210, indicating L1210 was responsible for an increase in ROS levels in MSCs. Moreover, the amount of mitochondrial transfer declined after the knocking down of NOX2 in L1210 (Fig. 1N). The results indicated that the ROS in MSC stimulated the transfer of mitochondria from MSC to L1210 cells. In our vivo imaging experiment, we concluded that NOX2 knockdown in L1210 indicated the lower homing process to the skull marrow after 24 h injection (Fig. 1O; Fig. S6D). These data indicated that mitochondria transfer from MSC to L1210 promote leukemia cells’ homing.

Here, using advanced animal live imaging and single cell-labelling, we showed that specific administered single MSC cell could direct leukemic cell homing in cell-to-cell contact dependent manner, probably through L1210 drived mitochondrial transfer. The present *in vivo* data at single-cell resolution may advance our understanding of the role of administered circulating MSCs in the development of hematologic tumor and also provide important guidance in the safe use of MSC-based therapies.

## CRediT authorship contribution statement

**Kunpeng Wu:** Conceptualization, Data curation, Formal analysis, Funding acquisition, Investigation, Methodology, Writing – original draft, Writing – review & editing. **Fen Wang:** Conceptualization, Data curation, Investigation, Methodology, Validation, Writing – original draft. **Min Shen:** Conceptualization, Data curation, Investigation. **Xiaohui Liang:** Writing – review & editing, Data curation, Validation. **Dan Wei:** Methodology. **Xi Zhu:** Methodology. **Ying Qu:** Methodology. **Xunbin Wei:** Conceptualization, Supervision. **Hua Jiang:** Conceptualization, Resources, Supervision. **Tong Chen:** Conceptualization, Funding acquisition, Resources, Supervision, Writing – review & editing.

## Ethics declaration

All animal procedures were approved by the Ethical Committee of Animal Experiments of the School of Pharmacy, Fudan University (2014-09-HSYY-CT-01).

## Conflict of interests

The authors declared no competing interests.
