# Supplementary material for: Bone marrow mesenchymal stroma cell serves as a harbor anchoring acute B lymphoblastic leukemia cells
Source: Genes Dis. 2025 Apr 12;12(6):101637. doi: 10.1016/j.gendis.2025.101637 (PMC12270788; doi:10.1016/j.gendis.2025.101637)
Supplement: Multimedia component 1 [file mmc1.docx]

**Bone marrow** **mesenchymal stroma cell serves as a harbor anchoring acute B lymphoblastic leukemia cells**

**Materials & Methods**

**Animals**

Balb/c mice aged 8 weeks were obtained from Shanghai laboratory animal center (Shanghai, China). All animals were housed and bred in pathogen-free conditions at the Laboratory Animal Center of School of Pharmacy affiliated with Fudan University (China).

**Cells**

Acute lymphoblastic leukemia cells (L1210) were purchased from Cell bank of the Chinese academy of sciences (China) and maintained in high-glucose DMEM with 10% FBS. MSCs derived from BM of C57BL/6 and Balb/c mice were purchased from Cyagen Biosciences Inc. (China) and maintained in low-glucose DMEM with 10% FBS. All experiments were conducted on MSCs at a passage 6 to 10.

**Cell labeling and transplantation**

Cells were labeled with fluorescent lipophilic tracers or fluorescent protein for cell tracking DiD and DiI (Invitrogen, USA), cell membrane dyes with uniform brightness and no toxicity, were used to label L1210 and MSC respectively. Cells were incubated in DiD or DiI for 50 min at 37 degree in the dark according to manufacturer instructions. L1210 were transfected by lentiviruses with U6-MCS-Ubiquitin-Cherry-IRES-puromycin (Genechem Co., China) at 6,000 viral particles per cell for 72 hours in cell culture medium. MSCs were transfected by adenoviruses with pDOV-mCMV-MCSEGFP (Obio Technology Co., ltd, China) at 5,000 viral particles per cell for 60 hours in cell culture medium. The transfection efficiencies of L1210s and MSCs were both above 85% based on conventional flow cytometry results. The labeled L1210s and MSCs were transplanted into mice by tail vein separately at a 20-minute interval.

**Mouse calvarium intravital microscopy (IVM)**

Mice were anesthetized by isoflurane inhalation. After being deeply anesthetized, the mouse scalp was incised to fully expose the marrow region, then fixed on the microscope stage stably with a heating pad. The vessels within the marrow can be labeled by 100μL FITC-Dextran (200 kDa, Sigma, Germany) at 10 mg/mL or by 50μL CD31-FITC antibody (Becton, Dickinson) via the tail vein of mice. 488 nm, 543 nm and 635 nm were chosen as the laser light, and the fluorescence signal was received at the wavelength of 500-520 nm, 580-610 nm and 650-700 nm respectively through a 16× water immersion objective lens (NA=0.8) or 20× water immersion objective lens (NA=0.8). The sequence scanning was performed to prevent the cross-staining between channels. After imaging, we disinfected and sutured the scalp incision of mice carefully for the next observations. The calvarium of the mice was imaged using A1R MP A1 (Nikon, Tokyo, Japan) or TCS SP8 (Leica, Germany). The images were analyzed using Imaris Software (Bitplane, Zurich, Switzerland).

***In vivo* flow cytometry (IVFC)**

The working principles and procedures of IVFC were also interpreted detailly in previous articles[^1^](#_ENREF_1). After being deeply anesthetized, mouse was positioned and fixed on the IVFC stage with a heating pad. An ear artery about 50-70μm in diameter was chosen under the illumination of 535 nm light emitting diode (LED). 543 nm and 633 nm laser light emitted continuously and was focused onto a slit across the chosen artery. Cell fluorescence was excited and then collected by the same microscope objective (40×, NA=0.6). The fluorescence signal was received at the wavelength of 580-610 nm and 650-700nm respectively.

**Conventional flow cytometry (FCM)**

Mice injected with labeled cells were euthanized. BM was collected from the hind limbs. Blood was extracted from the retro-orbital sinus of the mice. The cell suspensions were then incubated with Red Blood Cell Lysis Buffer (eBioscience, USA) at room temperature. After being washed with PBS, the packed cells were resuspended in PBS medium for analysis by conventional flow cytometry (FACS Aria II, Becton, Dickinson). Data analysis was carried out with FlowJo Software**.**

**Confocal laser scanning microscopy (CLSM)**

1×10^5^ GFP^+^ MSCs and 1×10^5^ mCherry^+^ L1210s suspended in medium were laid on the bottom of culture dish respectively. Then place the dish on the stage of CLSM (TCS SP5, Leica) equipped with a hood at condition of 5% CO_2_ and 37℃. 488 nm and 543 nm were chosen as the laser light, and the fluorescence signal was received at the wavelength of 500-520 nm and 580-610 nm respectively through a 40× oil immersion objective (NA=1.4). XYT and XYZ scanning model were applied to observe the dynamic living cell interaction between L1210 and MSC. The sequence scanning was performed to prevent the cross-staining between channels.

**L1210** **migration detection**

L1210s were cultured alone or cocultured with MSCs at a ratio of 1:1 for 2 days. Then L1210s were harvest to determine the cell migration abilities. The migration assay was performed as follows: 600μl DMEM with 10% FBS was added to the lower compartment of transwell chamber (Becton, Dickinson and Company, USA) containing 8μm pore. 300μl of the serum-free medium containing 2×10^4^ monocultured or cocultured L1210s were seeded to the upper compartment. After coculturing the upper and lower compartment in an incubator for 24 hours, we calculated the migrated cells to the lower compartment.

**Random spot generation**

The generation of random dots (RD) was referred to the article published in Nature[^2^](#_ENREF_2). RDs were generated and inserted within cell distribution region with the same amount of MSCs and given a diameter of 15 mm, similar to the observed average MSC diameter. The processed images were also next analyzed using Imaris Software (Bitplane, Zurich, Switzerland).

**AMD3100 CXCR4 blockade**

L1210 (5 x 10^6^/ml) were incubated in 250 ng/ml AMD3100 (Abcam, England) or control PBS in serum-free media for 1h at 37°C. Cells were then centrifuged and suspended for injection in mice.

**VLA4 blockage on L1210**

VLA4/α4 integrin (eBioscience, USA) were used to treat L1210 cells at 4 °C for 30 min at 10μg/ml. L1210 cells were washed for twice and suspended for injection in mice.

**RNA extraction and Real-time PCR**

L1210s were cultured alone or cocultured with MSCs at a ratio of 1:1 for 2 days. Then L1210s were harvest to extract total RNA using the TRIzol reagent (Thermo Fisher Scientific, USA). RNA was reverse-transcribed (RT) using Primescript RT reagent kit (Takara, Japan). PCR was performed using intron-spanning gene-specific primers (CXCR4: forward primer 5′-GACTGGCATAGTCGGCAATG-3′, reverse primer 5′-AGAAGGGGAGTGTGATGACAAA-3′; VLA-4: forward primer 5′-ATGCCAAATCTTGCGGAGAAT-3′, reverse primer 5′- TTTGCTGCGATTGGTGACATT-3′) and SYBR green master mix (Takara, Japan) on Step One Plus Real-time PCR System (Life Technologies, USA). Quantification was calculated using the 2-ΔΔCT method. The result is presented as fold change. β-actin (forward primer 5′- GGCTGTATTCCCCTCCATCG-3′, reverse primer 5′- CCAGTTGGTAACAATGCCATGT-3′) was used as an internal control.

**Mitochondrial transfer assay**

MSCs were stained with 200nM MitoTracker Deep Red FM (Yeasen, China) for 30min at 37°C. L1210 cells were stained to with 5μM 3,3-Dioctadecyloxacarbocyanine perchlorate (DiO, Yeasen, China) for 40min. Both of the two cell types were washed 3 times in PBS to remove the unbound probe before the directed co-culture. The stained L1210 were added to stained MSCs at a ratio of 5:1 for 24 hours. After incubation, images were acquired on a microscope (TCS, SP8 Leica). DiO and Deep Red Mitotracker were excited with 488 nm and 635 nm were chosen as the laser light for the detection of L1210s and mitochondria respectively. Mitochondrial transfer was quantified among L1210s by the mean fluorescence intensity (MFI) of Deep Red Mitotracker with flow cytometry.

**ROS assessment**

Cells were treated with N-Acetyl-L-cysteine (NAC, 5 mM, Beyotime Biotechnology, China), cytarabine (Ara-c, 200nM, Solarbio, China), or NAC (5 mM) and Ara-c (200nM) together. The ROS levels were detected using ROS Assay Kit (Beyotime, China) according to the manufacturer’s instruction. Briefly, cells were incubated with 10μM DCFH-DA solution for 30 min, and quantified by flow cytometry.

**Transfection of miRNA inhibitors**

Cells (3×10^5^ per well in a six-well plate) were cultured to reach a confluency of 60–80%. A mixture of NOX2 siRNA (50nM, Rubio Biotechnology, China, 5′- GACAGGAACCTCACTTTCCATA-3′) with Lipofectamine^TM^3000 Transfection Reagent (Invitrogen, USA) was applied to transfect L1210s according to the manufacturer’s instructions. After culturing for 24 h, the transfected L1210s were harvested.

**Statistical Analyses**

Data are presented as the mean ± SEM. Stata 10.0 software (Stata Corp, USA) was used for statistical analysis. T tests were applied to compare 2 groups and one-way ANOVA was applied to compare multiple groups with the Holm-Sidak test used for internal comparison between multiple groups. Data were considered significant if P<0.05.

**Supplemental figures and legends**

**
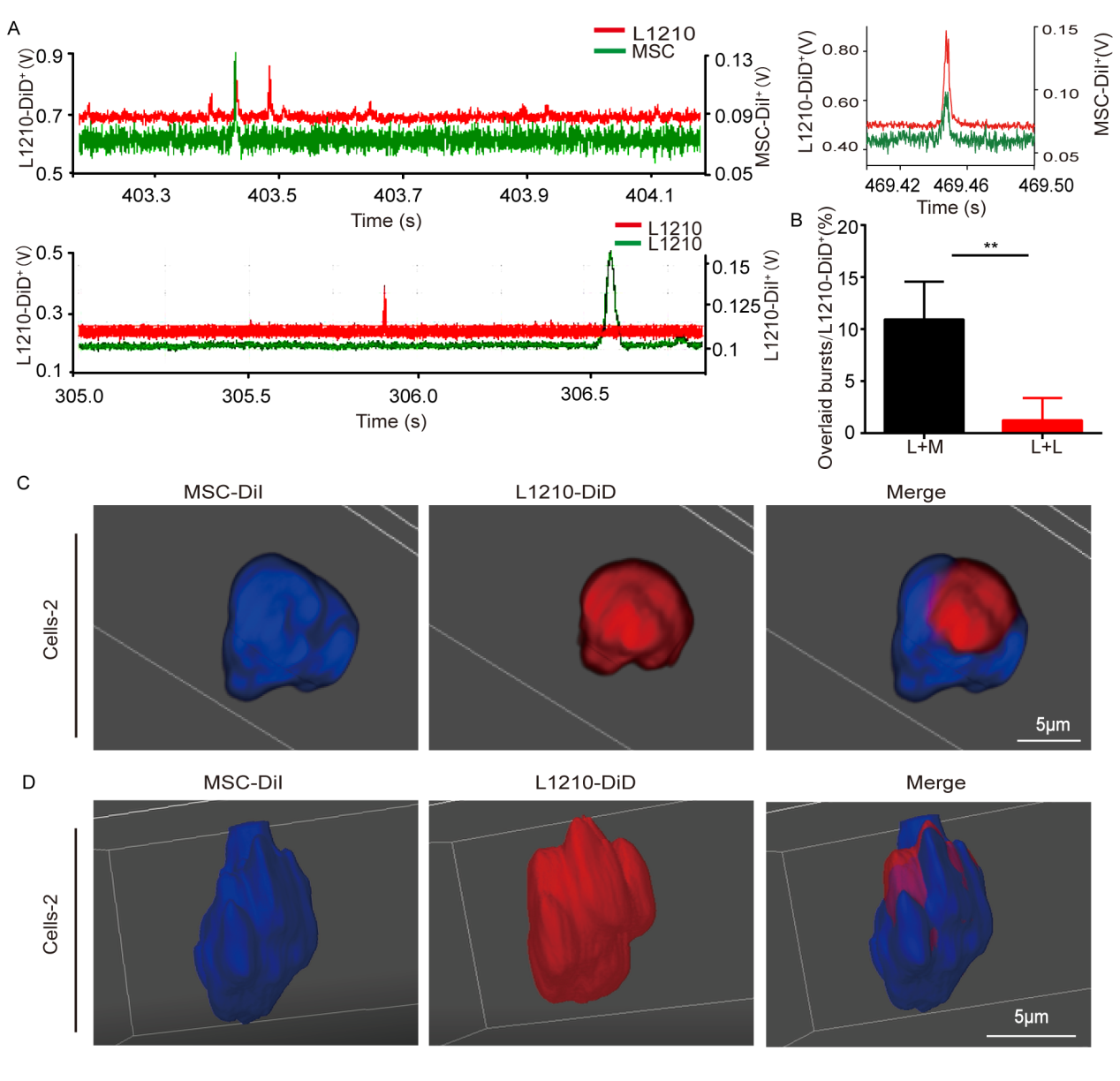
**

**Figure S1. L1210 cells and BM-MSCs were found to be co-localized in live murine circulation and BM.**

(A) Co-located cells were circulating together in blood under the IVFC. Red: L1210 labeled by DiD. Green: MSC labeled by DiI. (B) The proportion of co-localizing cells in PB under the IVFC (n=6). (C) The imaging of L1210+MSC co-localizing cells in PB under confocal. Red: L1210s labeled by DiD. Blue: MSC labeled by DiI. (D) The imaging of L1210+MSC co-localizing cells in BM of both hind limbs under confocal. Red: L1210s labeled by DiD. Blue: MSC labeled by DiI.

**
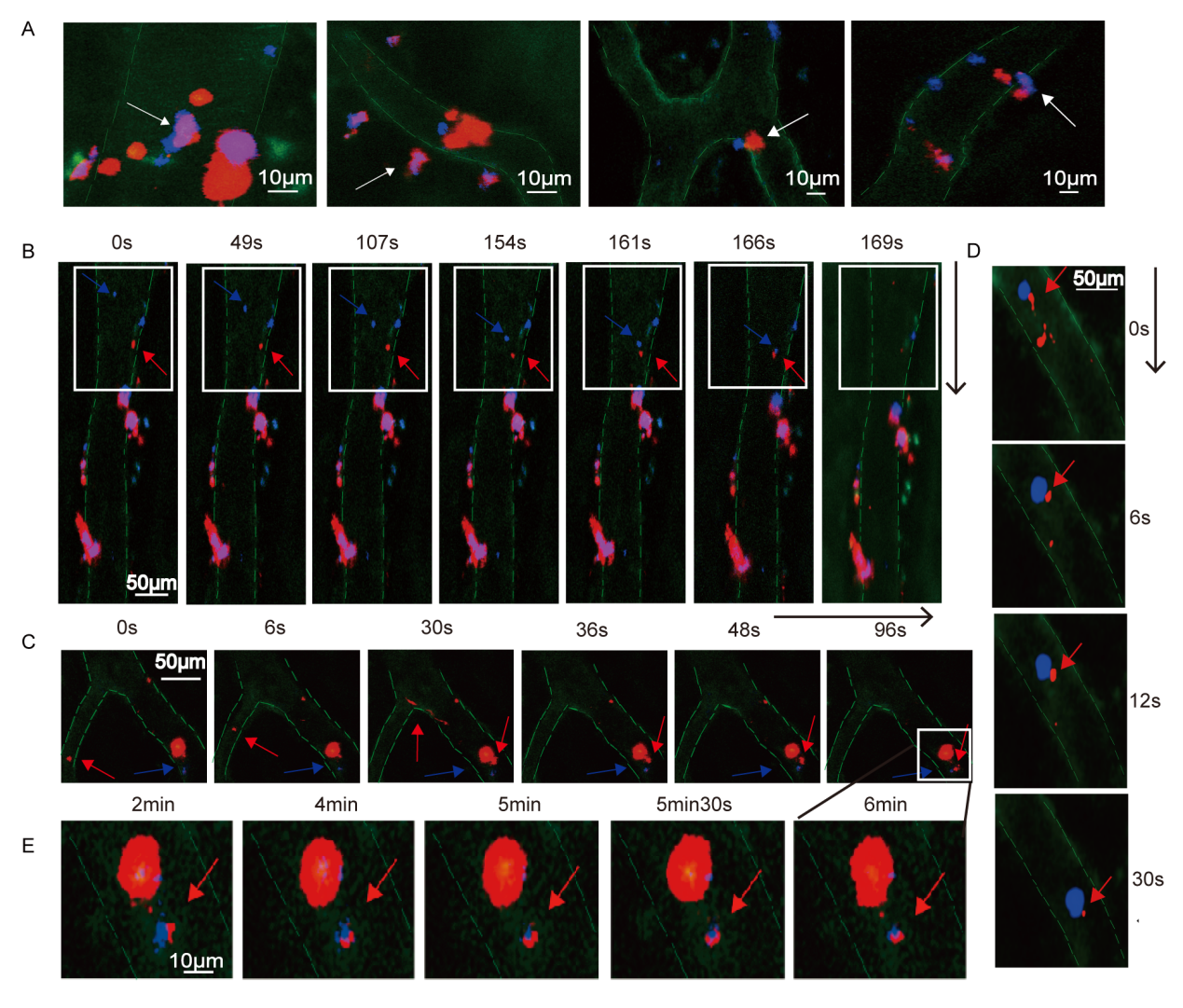
Figure S2. A motivator driven facing movement was found between L1210 cells and BM-MSCs.**

(A) Co-located cells distributed in skull marrow under the IVM. (B) MSCs were viewed to move towards L1210s gradually in bone marrow vessels. (C) L1210s were viewed to move towards to MSCs gradually in bone marrow vessels. (D) L1210 and MSC co-localizing cells travel together in the bone marrow vessels. (E) The subsequent stable adhesion of L1210 with MSC in vessels. (A-E) Red:L1210 labeled by DiD. Blue: MSC labeled by DiI. Green: bone marrow vessels labeled by CD31-FITC antibody.


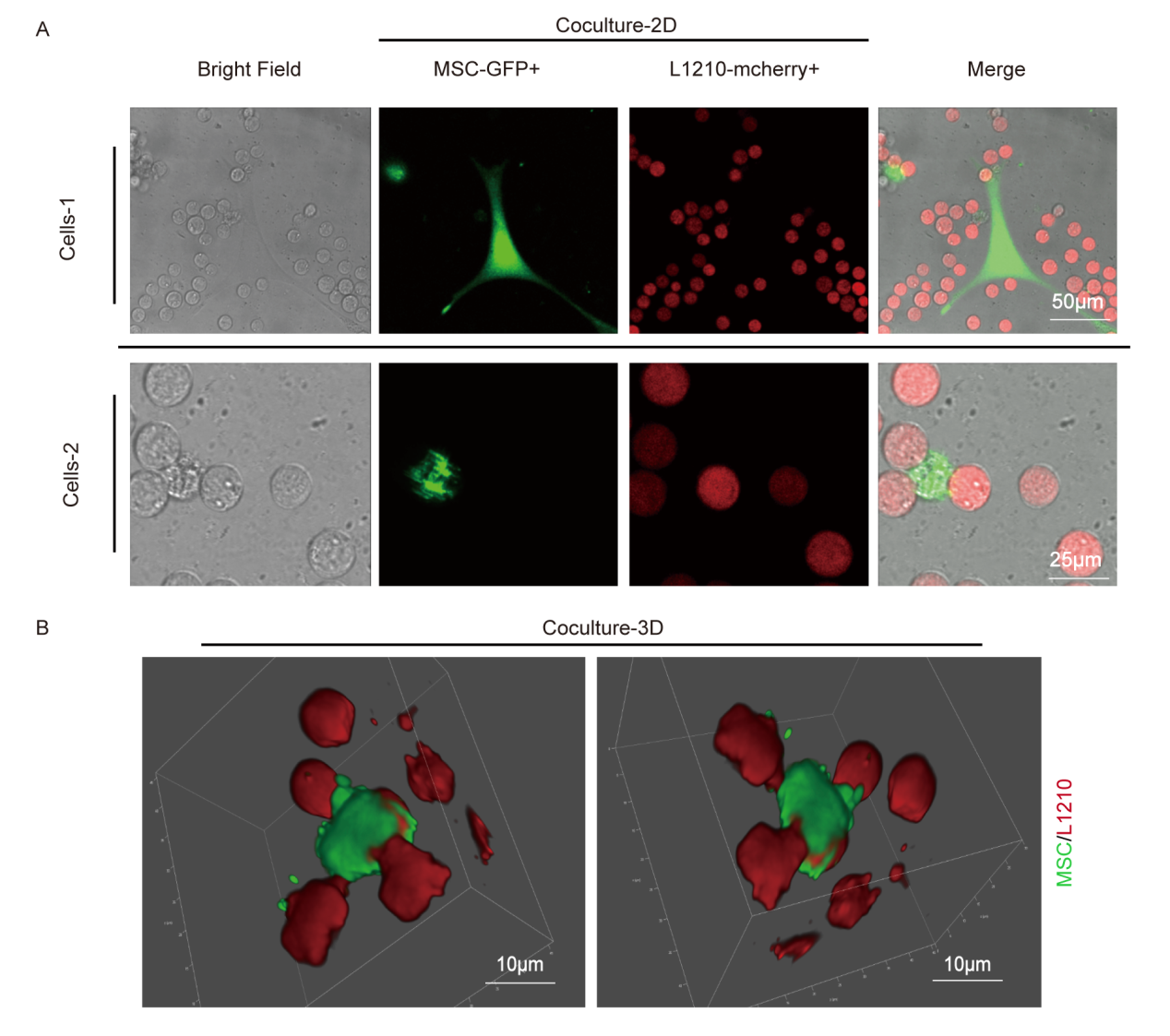


**Figure S3. The attraction and adhesion between L1210s and MSCs in vitro.**

The 2D imaging (A) and 3D imaging (B) of the mcherry^+^ L1210 and GFP^+^ MSCs after co-culturing under the confocal. Red: L1210s labeled by mCherry. Green: MSC labeled by GFP.


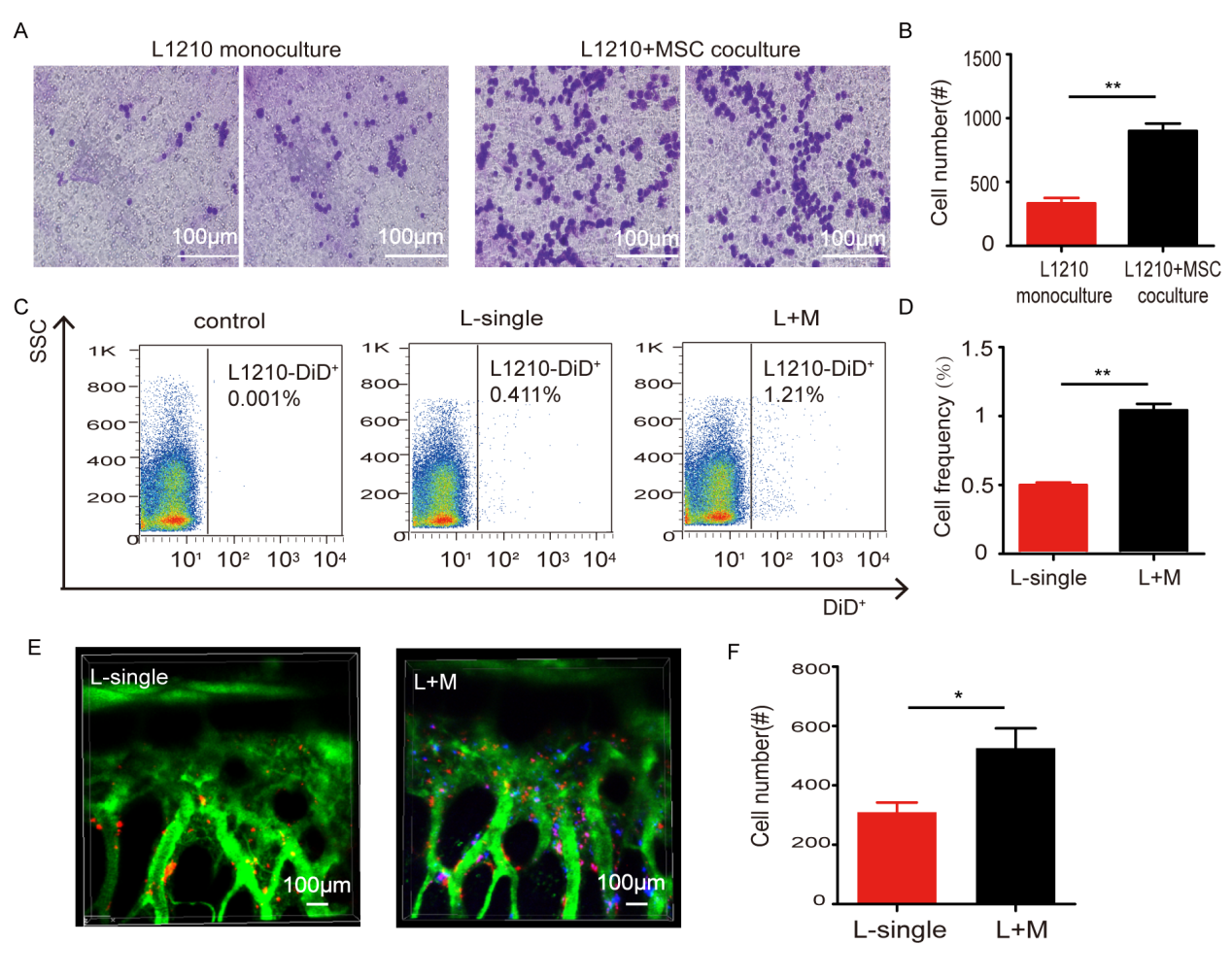


**Figure S4. BM-MSCs enabled L1210 cells homing into BM.**

(A-B) The transmigrating ability of L1210s pre-cultivation with BM-MSCs (n=5). The representative image of flow cytometry (C) and percentage of L1210 (D) in BM of both hind limbs in L1210 transplantation and L1210-MSC co-transplantation mice on day 1 after transplantation (n=5). (E) The *in vivo* imaging of the homing L1210s in skull marrow in L1210 transplantation group and L1210+MSC co-transplantation group. Red: L1210s labeled by DiD. Blue: MSC labeld by DiI. Green: bone marrow vessels labeled by FITC-dextran. (F) The stasitistic analysis of the homing L1210 cell number in the bone marrow per field (n=5).

**
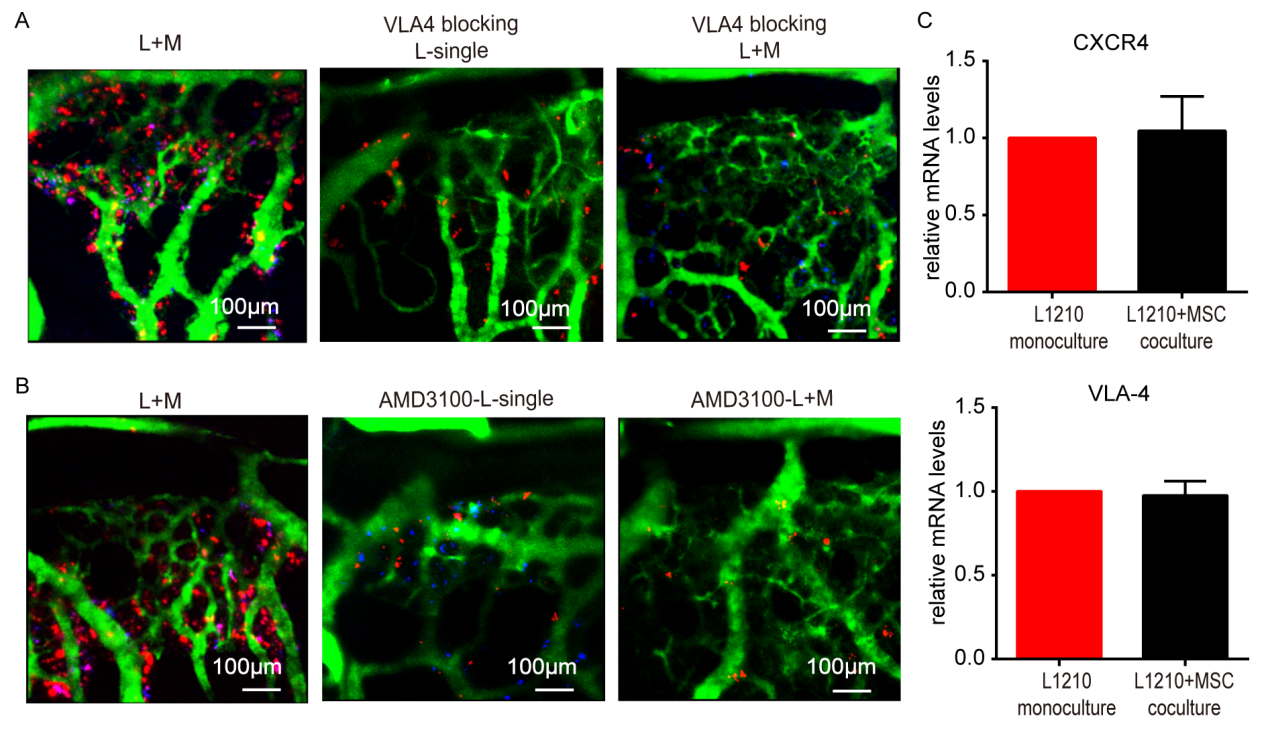
**

**Figure S5. Block of CXCR4 and VLA-4 on L1210 suppressed the pro-homing effect of BM-MSCs to leukemia cells.**

(A) The *in vivo* imaging of the homing L1210s in skull marrow in L1210-MSC, VLA-4 blocking L1210 and VLA-4 blocking L1210-MSC transplantation group. (B) The *in vivo* imaging of the homing L1210s in skull marrow in L1210-MSC, CXCR4 blocking L1210 and CXCR4 blocking L1210-MSC transplantation group. (A-B) Red: L1210s labeled by DiD. Blue: MSC labeld by DiI. Green: bone marrow vessels labeled by FITC-dextran. (C) Relative mRNA level of CXCR4 and VLA-4 in L1210 cells cultured alone and cocultured with MSCs (n=3).


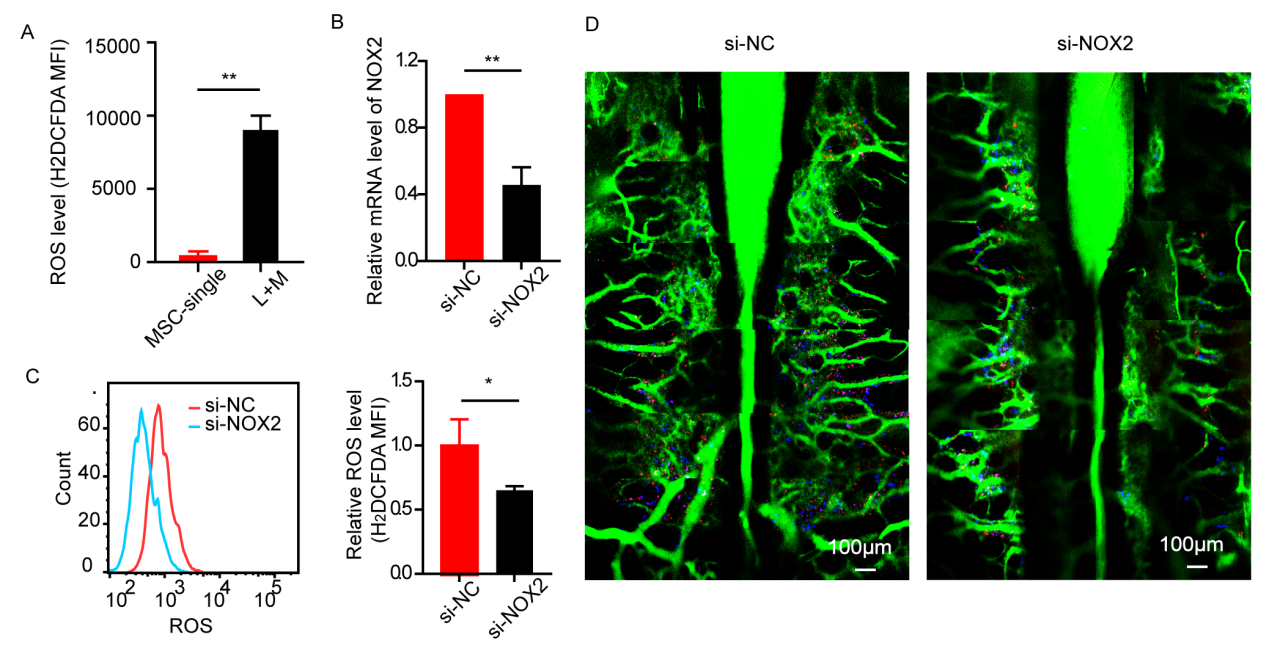


**Figure S6. Mitochondria transfered to L1210 via ROS-depended manner.**

(A) MSCs cultured alone and in coculture with L1210 cells were stained for ROS using H_2_DCFDA (10 mM) by flow cytometry (n=3). (B) Relative mRNA level of NOX2 in L1210. (C) ROS levels of MSCs co-cultured with si-NOX2 or si-NC L1210 for 24 h (n=3). (D) The *in vivo* imaging of the homing L1210s with the treatment of si-NC or si-NOX2 in skull marrow. Red: L1210s labeled by DiD. Blue: MSC labeld by DiI. Green: bone marrow vessels labeled by FITC-dextran.

**References**

1. Sipkins DA, Wei X, Wu JW, et al. In vivo imaging of specialized bone marrow endothelial microdomains for tumour engraftment. *Nature.* 2005;435(7044):969-973.

2. Acar M, Kocherlakota KS, Murphy MM, et al. Deep imaging of bone marrow shows non-dividing stem cells are mainly perisinusoidal. *Nature.* 2015;526(7571):126-130.

**Declaration of interests**

The authors declare that they have no potential conflict of interests.
